# Our Journey from the Study of Human Autoantibodies to the microRNA World

**DOI:** 10.3389/fimmu.2015.00110

**Published:** 2015-03-11

**Authors:** Kristianna M. Fredenburg, Edward K. L. Chan

**Affiliations:** ^1^Department of Oral Biology, University of Florida, Gainesville, FL, USA

**Keywords:** microRNA, autoantibody, GW body, endotoxin tolerance, IL-1beta

Our discovery of a novel cytoplasmic domain in HEp-2 cells recognized by the autoimmune serum of a patient led us on a journey into the world of microRNAs (miRNA). Our journey began with the identification and cloning of the novel glycine (G)–tryptophan (W)-rich protein, GW182, and its close interaction with the miRNA-binding protein, argonaute2 (Ago2). Discovering the important relevance of GW182 with miRNA function was the igniting force that steered us toward understanding the role of miRNAs in autoimmunity and the innate immune response. This brief paper highlights our journey and key findings.

## The Discovery of GW Bodies

**Figure 1:**
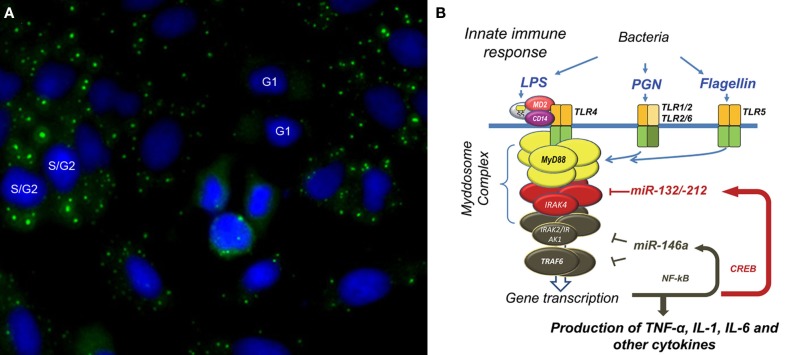
**Glycine (G)–Tryptophan (W) bodies (GWBs) and microRNA regulation in innate immune response**. **(A)** Using a human prototype serum, we identified a novel cytoplasmic domain (green) in HEp-2 anti-nuclear antibody assay; nuclei are counterstained blue with DAPI. We termed this GWB due to the presence of a 182-kDa glycine–tryptophan repeat-rich protein, GW182. Formation of GWBs is cell-cycle dependent with absence at G1 phase and an increase in size and number in late S/G2 phase. Increase in GWBs is seen in monocytes after LPS-stimulation. **(B)** Our proposed model of microbial tolerance and cross-tolerance regulated by miRNAs via targeting of Myddosome complex. Endotoxin tolerance in THP-1 cells primed and challenged with LPS involves the binding of LPS to the LPS-binding protein which is complexed to MD-2, CD14, and TLR-4. Binding results in formation of the Myddosome complex (MyD88, IRAK4, and IRAK 1/2), which then recruits TRAF6. These events lead to transcription of pro-inflammatory cytokines as well as miR-146a via NF-κB nuclear translocation. Upregulated miR-146a abrogates TNF-α transcription by blocking IRAK1/2 and TRAF6 mRNA expression even when challenged with high-dose LPS. This leads to a state of endotoxin tolerance. This phenomenon is observed with other microbial components – PGN and flagellin. However, PGN-mediated tolerance occurs through binding of PGN to TLR2, transcriptional activation of CREB, and a subsequent rapid induction of miR-132/-212. miR-132/-212 then inhibits IRAK4 leading to tolerance to PGN. Interestingly, upregulation of miR-132/-212 was also observed with cross-tolerance when cells were primed with PGN and subsequently challenged with TLR5-ligand, flagellin.

In the early 2000s, using the serum from a patient with motor and sensory polyneuropathy, we identified a 182-kDa autoantigen that localized to a novel cytoplasmic domain in HEp-2 cells (1). The cytoplasmic domain was distinct from other cytoplasmic organelles including lysosomes, endosomes, and the Golgi complex. The number and size of the foci were cell-cycle dependent with the largest and most abundant occurring during late S and G2 phase. Based on the predicted molecular size and the unique distribution of glycine (G) and tryptophan (W) repeats throughout the protein, the antigen was designated as GW182. The cytoplasmic entities/foci were thus termed GW182-protein-rich bodies or GW bodies (GWBs, Figure 1A).

The functional role of GW182 was explored with immunoprecipitation studies. We used human prototype anti-GW182 serum to identify a 100-kDa protein that co-immunoprecipitated with GW182 in HeLa cell lysate. The protein was identified, via mass spectroscopy, as Ago2. Ago2 is a member of the Argonaute family of proteins which bind miRNA and are involved in post-transcriptional gene silencing processes. Interestingly, we also identified autoantibodies to Ago2 as previously described anti-Su antibodies (2). Ago2, the only catalytic Ago protein, binds miRNA such that it is in position/configuration to recognize target mRNAs. Once the complex of Ago2–miRNA binds to the 3′UTR of target mRNA, GW182 is recruited to initiate translational repression and target mRNA degradation (3). Our discovery of GW182 as a key facilitator of Ago2–miRNA-mediated post-transcriptional gene regulation piqued our interest as to the functional role of miRNA in autoimmunity.

## miRNAs Associated with GW Bodies

MicroRNAs are now defined components of the large population of non-coding regulatory RNAs known to be transcribed in mammalian cells. With over an estimated 1000 miRNAs, their regulation of gene expression is diverse (4). For instance, a common mechanism of miRNA involves a single miRNA modestly repressing several different mRNAs in a common biologic process. Moreover, multiple miRNAs can cooperatively regulate individual components within a single molecular pathway. Intron-encoded miRNAs have been shown to regulate flanking mRNA expression and then modulate the same process (4).

MicroRNA expression is initiated by the transcription of a primary miRNA transcript in the nucleus (5). Cleavage of the transcript to a pre-miRNA by Drosha–DGCR8 microprocessor complex is followed by transport into the cytoplasm via exportin-5. Continual processing by protein Dicer generates the mature miRNA, which is about 20–23 nucleotides in length. Mature miRNA are loaded on Ago2, which allows for the right configuration to recognize potential mRNA targets in the cytoplasm. Translational control occurs when miRNA–Ago complexed with a target mRNA recruit GW182 to form the core miRNA-induced-silencing complex (miRISC). GW182 mediates deadenylation by interacting with the poly-A-binding proteins and recruiting deadenylases. Once the poly-A-tail is shortened, mRNA is degraded.

MicroRNA, Ago2, and GW182 are key components of GWBs. Other components include RNA decay factors and mRNA. Autoantibodies have been identified to components of GWB are predominantly directed to Ago2 and GW182 (6). As a matter of fact, autoantibodies have been detected in several autoimmune diseases including ataxia and motor and sensory neuropathy, Sjögren’s syndrome, systemic lupus erythematosus, primary biliary cirrhosis, and rheumatoid arthritis (RA) (7).

## GW Bodies in Biological Responses and as Marker of miRNA Activity

Our initial studies to examine the potential role of GWBs in a biological response involved stimulating human monocytes cell line (THP-1) with lipopolysaccharide (LPS). With LPS-stimulation, we noted a time-dependent increase in the number and size of GWBs with the highest number seen after 8 h LPS-stimulation as compared to unstimulated controls (8). We then evaluated a variety of chemokines and pro- and anti-inflammatory cytokines, for their ability to induce an increase in GWBs. Stimulation with pro-inflammatory cytokines, in particular TNF-α, resulted in a significant increase in GWBs as compared to unstimulated controls. When tested on peripheral blood monocytes (PBMCs) from a healthy donor, similar findings were observed. Thus, the increase in size and number of GWBs could be used as a marker for miRNA activity (8). About the same time, it was shown that LPS could stimulate the production of miR-146a, miR-132, and miR-155 in THP-1 cells. Thus, we proceeded to use THP-1 cells as a model to study miRNA expression and its relationship to GWB (8).

## miRNAs in Inflammatory Disease States

With the discovery of increased miRNA activity in pro-inflammatory states, we next turned our attention to miRNA expression in RA, a TNF-α-mediated autoimmune disease. The expression of several miRNAs was evaluated in PBMCs of RA patients and compared to healthy and disease controls. A statistically significant increase in miR-146a, miR-132, miR-155, and miR-16 mRNA levels was seen in PBMCs from RA patients. Furthermore, high levels of miR-146a and miR-16 correlated with active disease as defined by CRP and ESR values. Subsequently, the aberrant expression of miRNAs in RA has been underscored in several studies (5, 7). Overexpression of miR-221/222, miR-323-3p, and miR-203 has been noted in synovial fibroblasts in the arthritic phenotype of RA. Underexpression of miR-34a, miR-124a, and miR-23b was found to be related to increased synovial fibroblasts proliferation and their resistance to apoptosis (9). However, miR-146a is the only miRNA consistently reported elevated in other independent laboratories ranging from articular tissue to different blood cells of RA patients [reviewed in Ref. (5, 7)]. Together, these studies demonstrate the role of miR-146a in RA and suggested a pathogenic role in autoimmune diseases.

## miR-146a Regulates via NF-κB-Dependent Pathway

From the above discussed studies, miR-146a stood out as a potential key mediator of innate immune dysfunction with potential biological relevance in RA. It was demonstrated that LPS-induced miR-146a expression is nuclear factor-kappa B (NF-κB)-dependent. mRNA targets include IL-1 receptor-associated kinase 1 (IRAK1) and TNF receptor-associated factor 6 (TRAF6), which are key components of the Toll-like receptor (TLR)-4 signaling pathways. We took on the task of further defining the function of miR-146a in the innate immune response. First, we stimulated THP-1 cells with increasing doses of LPS and observed a dose- and time-dependent increase in TNF-α protein expression, peaking at 4–6 h. We then noted that MyD88 pathway adaptors, TRAF6 and IRAK1, mRNA expression, which also went up after LPS-stimulation with a profile similar to that of TNF-α. The expression of miR-146a was consistent with previous findings that miR-146a negatively regulates TRAF6 and IRAK1. The unexpected surprise, however, was the continued increase in miR-146a levels, reaching 30- to 100-folds by 72 h, even when the mRNA levels of TRAF6 and IRAK1 already returned to normal levels.

## miR-146a and Endotoxin-Induced Tolerance

Sustained high levels of miR-146a in the presence of LPS were postulated to play a dominant functional role in endotoxin tolerance. Endotoxin tolerance is the refractory response of neutrophils and macrophages to subsequent LPS-stimulation, leading to reduced production of pro-inflammatory cytokines. To explore this, THP-1 monocytes were primed with 0, 10, 100, or 1000 ng/ml of LPS for 18 h and then challenged them with 1000 ng/ml of LPS for 5 h. A 90% decrease in TNF-α protein and mRNA expression was seen with 10 ng/ml of LPS priming and a complete abrogation of TNF-α expression was seen at 100 and 1000 ng/ml of LPS. A concomitant decrease in TRAF6 and IRAK1 mRNA levels was also detected. In contrast, miR-146a mRNA levels continued to increase proportional to level of LPS. To demonstrate that miR-146a was critical for endotoxin-induce tolerance, two experiments were performed. Transfection of miR-146a mimic into THP-1 cells induced a comparable endotoxin tolerance state with statistically significant reduction in cytokines TNF-α, IL-1β, and IL-6 production when challenged with high-dose LPS, effectively mimicking LPS priming. One the other hand, transfection with a miR-146a inhibitor abolished LPS-tolerance even in THP-1 cells primed with low-dose LPS.

From these studies, the following molecular model was proposed to explain miR-146a mediation of LPS-tolerance (Figure 1B). LPS binds TLR-4–CD14 and another accessory protein MD-2. The LPS-initiated TLR-4 signaling cascade leads to the formation of the Myddosome complex (which includes MyD88, IRAK4, IRAK1/2), which then recruits TRAF6. This chain of events triggers activation and translocation of NF-κB and results in the transcription of immune-responsive genes and cytokines such as TNF-α as well as miR-146a. In essence, in our system, activation of the pathway leads to early TNF-α expression, then a steady increase of miR-146a which block IRAK1 and TRAF6 mRNA expression even when challenged with high-dose LPS, leading to the state of LPS-tolerance.

Since IL-1 signaling shares the same MyD88-dependent pathway, the relevance of miR-146a to IL-1β signaling was further examined (10). Unwarranted overproduction of cytokines, such as IL-1β, can cause severe pathological complications and thus elaborate mechanisms are needed to regulate its onset and termination. The capability of IL-1β to induce tolerance and cross-tolerance against various inflammatory ligands was investigated. IL-1β-stimulated THP-1 monocytes showed a gradual increase of miR-146a, reaching 15-fold expression by 24 h. miR-146a upregulation induced tolerance toward subsequent challenges of IL-1β, LPS, peptidoglycan (PGN), Pam, and flagellin in THP-1 cells. The induction of tolerance was dependent on IL-1β-priming dose and associated increase of miR-146a expression. Thus, miR-146a seems to operate as a negative regulatory feedback mechanism to prevent the destructive consequences of uncontrolled cytokine or chemokine production during the IL-1β and TLR signaling cascade. Given that the miR-146a response by IL-1β was observed in primary mouse macrophage as well as in human monocytes, miR-146a is now demonstrated to play an important physiological role in the regulation of IL-1β signaling (10).

In support of our findings, several *in vivo* studies using miR-146a null mouse model have established miR-146a as a negative regulator of inflammation and a key player in hematopoietic malignancies. Lack of miR-146a in these mice leads to hyperresponsiveness of macrophages to endotoxin and an exaggerated inflammatory response in endotoxin-challenged mice (11, 12).

## TLR2/TLR5-Induced miR-132/-212 Functions in Tolerance and Cross-Tolerance

miR-146a may not be the only miRNA involved in microbial tolerance. When we primed THP-1 cells with PGN, similar to LPS-stimulation, exposure of THP-1 cells to PGN, led to an early increase in TNF-α protein expression and a decline within 24 h. When cells were primed with PGN and subsequently challenged with PGN, tolerance was observed. We determined that unlike LPS-induced tolerance, PGN-induced tolerance occurs through rapid induction of miR-132/-212 via CREB transcription factor. Induction of miR-132/-212 leads to inhibition of IRAK4 mRNA expression. Furthermore, instead of TLR-4-mediated activation, activation is mediated through TLR2. Cross-tolerance was observed when cells primed with PGN were subsequently challenged with TLR5-ligand, flagellin. Collectively, these data demonstrate that miRNAs target components of the Myddosome complex in order to induce tolerance/cross-tolerance (13). It should be noted that in an earlier study using miR-132/-212 knockout mice, loss of the miRNA cluster did not apparently impact LPS-induction of specific plasma cytokine levels, including TNF-α (14). This is not inconsistent with our findings in THP-1 monocytes that the role of miR-132/-212 in tolerance and cross-tolerance was primarily induced from TLR2 ligand PGN or PAM (13).

Our studies of miRNAs in innate immunity and tolerance, demonstrates that there are a subset of miRNAs with dominant functions (9). A dominant miRNA is best defined as a miRNA whose presence or absence dictates cellular function. When induced, it can affect many target genes in a dose-dependent manner. miR-146a is the prototype in innate immune response. In adaptive immunity, miR-182 has been proposed to act in this same dominant manner. *In vivo* and *in vitro* studies show that miR-182 induction by IL-2 promotes clonal expansion of T helper cells through Foxo1 regulation. Blocking miR-182 can lead to improvement of arthritis in the ovalbumin-induced arthritis model. Dominant miRNAs if clinically relevant may provide therapeutic targets for autoimmune diseases (15).

## The Voyage Continues

In summary, our observation, over 10 years ago, of a novel cytoplasmic entity introduced us to an important concept in gene regulation. The identification of miRNAs in autoimmune disease provided us with the foreground to explore their function. Our studies have provided important mechanistic insight into their regulation of the innate immune response. It is exciting to think that we may have new targets to assist in diagnosing, monitoring, and possibly treating diseases that are impacted by the dysregulation of innate immunity. Continual exploration and collaborative efforts to explore how these master regulators are altered during inflammatory disease processes may be the key to uncovering the etiology of immune-mediated diseases.

## Conflict of Interest Statement

The authors declare that the research was conducted in the absence of any commercial or financial relationships that could be construed as a potential conflict of interest.
